# Fibromyalgia: Understanding, Diagnosis and Modern Approaches to Treatment

**DOI:** 10.3390/jcm14030955

**Published:** 2025-02-02

**Authors:** Tamara Filipovic, Aleksandar Filipović, Dejan Nikolic, Francesca Gimigliano, Jelena Stevanov, Marija Hrkovic, Ivana Bosanac

**Affiliations:** 1 Institute for Rehabilitation, 11000 Belgrade, Serbia; tamarabackovic@gmail.com (T.F.); hrkovicm@yahoo.com (M.H.); 2 Faculty of Medicine, University of Belgrade, 11000 Belgrade, Serbia; aleksandar.filipovic11@gmail.com (A.F.); denikol27@gmail.com (D.N.); 3 Center for Radiology, Faculty of Medicine, University Clinical Centre of Serbia, University of Belgrade, 11000 Belgrade, Serbia; 4 Department of Physical Medicine and Rehabilitation, University Children’s Hospital, 11000 Belgrade, Serbia; 5 Department of Physical and Mental Health and Preventive Medicine, Luigi Vanvitelli University, 80138 Naples, Italy; francescagimigliano@gmail.com; 6 Clinic for Rehabilitation Dr M. Zotović, 11000 Belgrade, Serbia; jejastevanov@gmail.com

**Keywords:** fibromyalgia, pathophysiology, diagnostic criteria, patient-centered therapy

## Abstract

Fibromyalgia (FM) is a chronic condition characterized by generalized musculoskeletal pain associated with other symptoms, especially sleep and mood disorders, fatigue, and cognitive dysfunctions. The etiopathogenesis of FM is not sufficiently known, and regardless of numerous research, the clinical presentation is nonspecific, which makes it difficult to approve a timely diagnosis and, subsequently, an adequate therapeutic approach. Genetic, hormonal, immunological, and environmental factors are cited as potential factors in the development of this condition. Diagnosis is based on a clinical approach and known diagnostic criteria, while additional methods, such as radiographic, magnetic resonance, or laboratory analyses, can be useful to exclude other conditions. The heterogeneity of FM significantly impacts both diagnosis and treatment, as it presents a wide spectrum of symptoms that vary in severity, combinations, and underlying contributing factors. This variability is a challenge for clinicians and requires a holistic, comprehensive, multidisciplinary, patient-centered approach. According to The European League Against Rheumatism (EULAR) from 2016, treatment begins with patient education and involves the simultaneous application of pharmacological and nonpharmacological treatments. The application of only pharmacological or nonpharmacological treatment is most often not successful. Due to differences in pain threshold, psychological factors, and comorbidities, patients may respond differently to the same interventions. Although there is no universal treatment, this review brings up the fact that the timely recognition of symptoms and a tailored treatment with a patient-centered plan can significantly improve the quality of life of patients.

## 1. Fibromyalgia

Fibromyalgia (FM) is a chronic disorder characterized by a wide range of symptoms: widespread pain, fatigue, sleep disturbances, including also cognitive dysfunction, anxiety, depression, and emotional difficulties, which are accompanied by an inability to perform usual daily activities. Estimates suggest that this syndrome affects between 2% and 8% of the world’s population, primarily working-age people, and it is significantly more common in women than in men, in a ratio of 3:1 [1]. The causes of fibromyalgia are still not fully understood; there is no single factor cause of FM, it is rather believed to be a combination of genetic predisposition, immunological and neuroendocrine dysfunctions, and environmental factors, especially psychological or physical stressors, but for now there is no consensus regarding etiopathogenesis, classifications or treatment [1,2].

Fibromyalgia is often described as a pain processing disorder in the central nervous system, which makes patients hypersensitive to pain. Elevated levels of certain neurotransmitters, such as glutamate and substance P, may contribute to the sensitization of pain signals. Research shows that fibromyalgia can be associated with a disorder in the functioning of mechanisms like relaxation and stress, as well as with abnormalities in hormonal levels.

Dysfunction of monoaminergic transmission is currently the main observed change in FM, which leads to increased levels of the excitatory neurotransmitters glutamate and substance P and decreased levels of serotonin and norepinephrine in the spinal cord at the level of descending antinociceptive pathways. In addition to this dysfunction, the dysregulation of dopamine, as well as the altered activity of endogenous opioids, have been observed, which all together explain the central origin of the physiopathology of FM [2]. Peripheral pain generators have been the subject of researchers as the relevant cause of fibromyalgia in recent times. In this case, patients manifested symptoms such as cognitive impairment, chronic fatigue, sleep disturbance, intestinal irritability, interstitial cystitis, and mood disorders. Peripheral abnormalities can contribute to an increase in nociceptive tone in the spinal cord, which leads to central sensitization [3].

Regardless of the abundance of available information, several gaps in the existing literature about FM continue to challenge researchers and clinicians in understanding and managing the condition effectively. These gaps cover diagnostic challenges, limited understanding of pathophysiology, and the role of neuroinflammation. Although FM predominantly affects women, there is also limited research on how the condition manifests and progresses in men. Research on the efficacy of combining pharmacological and nonpharmacological treatments is still limited. Furthermore, research often focuses on symptom reduction rather than improvements in the quality of life, functioning, or patient satisfaction.

This review offers a critical and detailed analysis of the diagnosis, heterogeneity of fibromyalgia, evolution of diagnostic criteria since 1990, and treatment of fibromyalgia. Additionally, this review synthesizes various hypotheses regarding the aetiopathogenesis of fibromyalgia and emphasizes challenges placed in front of patients and clinicians in the clinical practice. The highlight here is an individualized, patient-centered approach to care. Clinicians need training to recognize the various presentations of FM and address patient concerns about their conditions. They always have to bear in mind that patients who feel validated, listened to, and supported by their clinicians are more likely to adhere to treatment. Dismissive or judgmental attitudes from clinicians can lead to mistrust.

### 1.1. Pain Mechanisms—Central Sensitization

Aberrant signal processing in the peripheral and central nervous system is the basis of peripheral and central sensitization, both of which maintain chronic pain. Neuroinflammation plays a key role in the development of sensitization and is characterized by the activation of glial cells and the production of proinflammatory chemokines and cytokines in the central and peripheral nervous system. Various clinical studies using functional magnetic resonance have confirmed central neuronal changes in nociceptive processes. It was back in 2004 when Gracely et al. [4] showed that after the same strength of pressure stimulus, patients with FM have greater activation in the areas of the brain that process pain compared to the control group of subjects, primarily in the posterior insula and the secondary somatosensory cortex [5]. In the same areas of the brain, other studies indicated a change in the level of several key neurotransmitters and modulators, which, depending on the central nervous system (CNS) site and receptor, have either inhibitory or excitatory effects [5,6]. One of the most important neurotransmitters is Glutamate, which plays a key role in excitatory and sensitizing effects as well as in nociception while expressing some inhibitory effects in descending pain pathways. It is suggested that an optimal glutamate tone is necessary, whereby too little or too much can cause the activation of hypothalamic-pituitary-adrenal axis (HPA) [7].

An increased glutamate level within the insula and posterior cingulate was observed in patients with FM [8,9].

There is an important neuropeptide that coexists along with glutamate, substance P (SP), which causes the sensitization of the dorsal horn neurons and is found in the primary nociceptive afferents [9,10]

The sensitivity to pain increases alongside the level of SP. Several studies have demonstrated that the concentration of SP in the cerebrospinal fluid (CSF) of patients with FM was significantly elevated in the amounts of two to three times higher than in healthy subjects [11,12,13,14].

Another crucial, most prevalent inhibitory neurotransmitter involved in FM symptoms is gamma-aminobutyric acid (GABA). It amounts to 60% to 70% of all synapses in the central nervous system and is found in high concentrations in all brain regions and spinal cord. Its excess and deficiency may contribute to FM symptoms and play a key role in sensitivity for movement disorders, pain, sleep, mood, and cognition [11,12].

Serotonin (5-hydroxytryptamine, 5-HT) is also an important and well-known monoamine neurotransmitter, with an important role in many biological and behavioral processes such as the inhibition of pain pathways, mood, cognition, sleep, digestion, wound healing, reproductive activity, and circadian rhythms. In patients with FM, decreased levels of serotonin were observed compared to normal controls and were associated with increased pain sensitivity, fatigue, and depression. This decrease in serotonin may also participate in the development of FM [15]. Similarly, dopamine (3-hydroxytryptamine) has been found to be involved in the descending inhibitory modulation of pain in the brain, as well as in body movements and coordination, motivation, and rewards. A key role of dopamine in the etiopathogenesis of fibromyalgia is shown in its pain regulating capability. Altered levels of these neurotransmitters may be one of the causes of FM onset. Seo et al. [16] used [C]-(R)-PK11195, a ligand of positron emission tomography (PET) for a translocator protein (TSLO), expressed by activated microglia or astrocytes, to identify specific brain regions (left and right postcentral gyrus, left precentral primary motor cortex, left superior parietal gyri, left medial frontal, left superior frontal, primary somatosensory cortex, left superior parietal gyri, and left precuneus) exhibiting abnormal neuroinflammation levels in patients with fibromyalgia, and complex regional pain syndrome (CRPS). Elevated neuroinflammation in the primary somatosensory cortex may contribute to central sensitization due to excessive neuroinflammation stimulating nociceptors. Similarly, dysfunction and heightened neuroinflammation in the primary motor cortex may impair the descending pain pathway and result in inadequate pain modulation. Increased neuroinflammation in the left frontal regions appears to play a role in the neuropathology and cognitive impairment observed in FM and CRPS patients. Abnormal neuroinflammation in the left precuneus might be linked to trauma experienced by individuals with these conditions. Notably, their study found that FM patients demonstrated greater neuroinflammation in the precentral and postcentral gyrus compared to CRPS patients and healthy controls [16].

Decreased cortical thickness has been reported in specific parts of the brain (such as the right superior and right and left middle temporal gyrus, left superior frontal gyrus, right fusiform gyrus, and the left amygdala) in patients who reported symptoms of fibromyalgia compared to healthy subjects. These findings are accompanied by reductions in gray matter volume in the prefrontal cortex, the amygdala, and the anterior cingulate cortex [16,17].

The same study [16] established a connection between higher levels of affective pain and increased neuroinflammation in the left medial frontal, left superior parietal, and left amygdala regions in FM patients. Given the relationship between elevated stress and Post-Traumatic Stress Disorder (PTSD) with heightened neuroinflammation in FM, psychological trauma and stress are suggested as potential primary drivers of neuroinflammation and pathological symptoms in FM [16,18,19].

### 1.2. Pain Mechanisms—Peripheral Sensitization

Nociceptors (unmyelinated C fibers and myelinated Aδ fibers) are the primary afferent neurons involved in the detection and encoding of noxious stimuli, transduction of noxious information via cell membranous ion channel (transient receptor potential (TRP), and voltage-gated ion channels) into afferent action potentials, and finally, in the transmission of pain signals to the CNS. TRP channels play a key role in the development of hyperalgesia as they can be sensitized by inflammatory mediators [20,21,22,23,24]. Researchers have identified the role of specific TRP channels, such as TRP ankyrin 1 (TRPA1), in the mediation of prolonged hypersensitivity, which makes it a potential therapeutic target in analgesia [21]. Moreover, they can synthesize and release neuropeptides such as substance P, Calcitonin gene-related peptide (CGRP), neurotransmitters, and inflammatory mediators. All these mediators can amplify the local inflammatory response, acting on other ‘silent’ C fibers to lower their activation threshold and increase the excitability of their neurons. This leads to peripheral sensitization [23,24,25,26,27].

The peripheral origin of FM has been of interest to researchers over the past decade. The presence of small fiber neuropathy (SFN—a peripheral polyneuropathy that, like FM, causes chronic widespread pain, exertional intolerance, gastrointestinal symptoms, and chronic headache) has been documented in about half of FM patients by differently designed studies [28,29,30,31,32]. Electrophysiologists report excess, spontaneous, and prolonged firing of C and Aδ pain fibers in both adults with FM and SFN, as well as electrical hyperactivity and distal degeneration of the peripheral neurons that mediate pain, heat and cold sensation, and internal autonomic functions [30,31,32,33].

Considering that skin biopsy is an invasive procedure that, therefore, can only be offered to a limited number of patients with FM, imaging techniques, such as ultrasound (US), could play a role in the diagnosis of SFN.

A study by Di Carlo et al. [34] investigated in detail the ultrasound (US) abnormalities, expressed in terms of CSA (cross-sectional area) and power Doppler signal, between the nerves of FM patients and healthy controls. Results of the study demonstrated that FM patients tend to show a larger CSA than healthy subjects, and the differences are significant at multiple sites, involving both peripheral and cranial nerves, especially the sural nerve, the sixth cervical nerve root, and the vagus nerve, bilaterally [34].

## 2. Pathophysiology

### 2.1. Genetic Factors

Numerous studies support the theory that genetic factors can predispose to the occurrence of FM, as well as that significant risk factors are the polymorphisms of genes involved in mood disorders [35,36]. Serotonin (5-hydroxytryptamine [5-HT]) plays a significant role in pain regulation as a key neurotransmitter. The serotonin transporter (5-HTT) is crucial for regulating serotonin levels by facilitating its reuptake from synapses into presynaptic neurons, thereby ending extracellular signaling. In humans, 5-HTT is encoded by the SLC6A4 gene, one of the most extensively studied genes in neurobiology. This gene includes a polymorphic region, the 5-HTT-linked polymorphic region (5-HTTLPR), characterized by a 43-base pair insertion/deletion [37]. This functional polymorphism (5-HTTLPR) in the 5′ regulatory region of the SLC6A4 gene comprises two primary alleles, a short- S and a long- L allele. The S allele is associated with a decreased transcription of the 5-HTT gene, resulting in lower 5-HTT expression, which is linked to an increased sensitivity to pain, emotional stress, and depression hallmarks of FM. Numerous studies have shown that stressful live events or chronic stress can exacerbate the serotonergic dysregulation caused by these polymorphisms, amplifying the risk of FM development and intensifying symptoms such as mood disorders, hyperalgesia, and fatigue [35,36]. The S allele is more prevalent in Asian populations (79%) compared to Western populations (42%), while the L allele is more commonly observed in individuals of Caucasian origin [36]. Except for the abovementioned serotonin transporter (SLC64A4), numerous genes are potential candidates for the development of FM. One such gene is the transient receptor 2 potential vanillin channel gene (TRPV2), which is mapped to chromosome 17q11.1-q12.12 [35,36,37]. This gene encodes a channel that plays a key role in nociception. Polymorphisms in TRPV2 may alter its function, affecting pain processing in FM. Environmental factors such as injury, physical trauma, inflammation, and thermal stress can activate the TRPV2 gen, which, if dysregulated, can lead to altered nociceptive signaling and central sensitization, increased pain severity, and discomfort in FM patients [36,37].

A study by Nugraha et al. [38] has shown that brain-derived neurotrophic factor (BDNF) expression is increased in patients with FM both in plasma and serum, as well as cerebrospinal fluid, compared to healthy subjects. The same study has shown results that suggest that BDNF polymorphisms (rs7124442 and rs2049046) are associated with body mass index and anxiety, respectively, in patients with FM. Significant differences were not found in a subgroup with depression. Other gene polymorphisms, considered potential genes for FM, are the 2A (5-HT2A) receptor, catechol-O-methyltransferase (COMT), and dopamine receptor (DRD3) [32,33]. No correlation between the COMT val158Met polymorphism and fibromyalgia and the 5-HTTLPR S/L allele was found, as per metanalysis by Lee et al. [39]. Their metanalysis failed to show an association of the 5-HTTLPR S/L polymorphism with fibromyalgia susceptibility but found an association of the 5-HT2A receptor 102T/C polymorphisms with fibromyalgia susceptibility [40,41]. Despite the potential relevance of these functional polymorphisms to fibromyalgia [41], this metanalysis failed to detect a significant association between the COMT val158Met polymorphism and the 5-HTTLPR S/L polymorphism with fibromyalgia susceptibility. Polymorphism of the Dopamine receptor D3 -ser9Gly seems to be associated with diffuse noxious inhibitory control efficiency in FM patients compared with control [42].

### 2.2. Hormonal Changes

Several hormonal systems dysregulation plays a significant role in the development and maintenance of FM. The hypothalamus-pituitary-adrenal (HPA) axis plays a central role in the body’s stress response and has been implicated in the development of certain fibromyalgia (FM) symptoms. These symptoms are influenced by the HPA axis’s end product, cortisol. Although there is no clear consensus regarding changes in plasma cortisol levels in FM patients, disruptions in its circadian rhythm are frequently reported. A study by Crofford et al. observed reduced daily fluctuations in cortisol levels and a flattened diurnal cortisol curve in FM patients compared to healthy controls [43,44]. Additionally, decreased cortisol secretion in response to adrenocorticotropic hormone (ACTH) tests has also been documented [45]. Dysregulated cortisol contributes to widespread pain, sleep disturbances, and fatigue, exacerbating the impact of environmental and psychological stressors. On the other hand, chronic stress can further desensitize the HPA axis, creating a feedback loop of worsening symptoms [46]. Worsening of FM symptoms due to stress has been widely documented from an epidemiological point of view, particularly through clinical questionnaires [43]. The potential involvement of the growth hormone (GH)/insulin-like growth factor 1 (IGF-1) axis in FM has also been explored [47,48,49,50,51,52]. Since the half-life of GH is too short for effective measurement, IGF-1 levels are used as an indirect marker of GH secretion. Atamer et al. found lower levels of IGF-1 and GH in FM patients and reported elevated leptin levels compared to controls. Their study also highlighted a correlation between higher leptin levels and increased pain intensity (as measured by the visual analogue scale (VAS)), body mass index (BMI), tender point sensitivity, and disease duration [48]. Conversely, research by Tander et al. did not observe significant differences in IGF-1 and GH levels in FM patients [49]. As GH secretion primarily occurs during stage 3 sleep—and considering that 80% of FM patients experience sleep disturbances—it remains uncertain whether these hormonal changes are a cause or consequence of FM [53,54].

Reduced GH secretion contributes to increased muscle pain and weakness, reduces the ability to recover from physical stress, and nonrestorative sleep, which further reduces GH secretion, creating a vicious circle of fatigue [53]. Dysregulation of sex hormones, especially estrogen, considering the higher prevalence of fibromyalgia in the female population, was investigated. Some studies have shown that FM symptoms often worsen during the time of hormonal shifts (menstrual cycle, pregnancy, menopause), thus manifesting an increased pain sensitivity, fatigue, and mood disorder [55].

It was also found that hormonal dysregulation directly interacts with neurotransmitter systems implicated in FM, such as serotonin, dopamine, and norepinephrine, and leads to increased severity of FM symptoms [56]. On the other hand, the results of other studies suggest that this role is limited, with the most notable result being an elevated serum concentration of G protein-coupled estrogen receptor (GPER) in fibromyalgia patients compared to healthy controls. The hypothesis of the possible use of this receptor as a potential diagnostic biomarker based on its strong association with FM can be put forward; however, the precise pathophysiological mechanism remains unclear [57]. Understanding hormonal dysregulation in fibromyalgia is unnecessary for tailoring treatment for FM.

### 2.3. Immune System and Neuroinflammation

A growing body of evidence supports the involvement of neuroinflammation in the peripheral tissues, spinal cord, and brain in the pathophysiology of fibromyalgia (FM) [58,59,60,61,62,63,64]. Neuroinflammation arises from the activation of glial and mast cells and the release of biologically active substances, such as chemokines and cytokines, which trigger both innate and adaptive immune responses. FM patients exhibit elevated serum levels of inflammatory interleukins (IL), including IL-6, IL-8, IL-1β, IL-33, and tumor necrosis factor-α (TNF-α). A meta-analysis conducted by O’Mahony et al. [62] demonstrated that FM patients have significantly higher levels of proinflammatory cytokines, such as TNF-α, IL-6, and IL-8, as well as the anti-inflammatory cytokine IL-10, in their peripheral blood compared to healthy controls. This highlights the potential role of cytokines in FM pathogenesis. Proinflammatory interleukins, such as IL-1β and IL-6, contribute to increased pain sensitivity by promoting inflammation and driving both central and peripheral sensitization. The presence of these cytokines in chronic pain conditions can also lead to the overexpression of N-methyl-D-aspartate (NMDA) and α-amino-3-hydroxy-5-methyl-4-isoxazolepropionic acid (AMPA) receptors, which are implicated in the mechanisms underlying nociplastic pain [58]. Some authors have gone a step further (Mendieta et al.) [61], emphasizing the important role of neuroinflammation and, in particular, certain interleukins, such as IL-6 and IL-8—in the clinical presentation of FM [61,62,63,64]. The authors pointed out that monitoring their levels could be correlated with the severity of FM. Due to this increased neuroinflammatory response, glial cells are activated, which are responsible for central sensitization through the excitatory glutamate effects. A connection with stress has also been suggested due to the dependence of IL-8 synthesis on sympathetic stimulation. Targeting neuroinflammation could address underlying mechanisms, offering a more fundamental approach to treatment, contrary to the current FM treatments, which focus primarily on symptoms. Considering that the studies have shown increased glial activation and level of proinflammatory cytokines, drugs that inhibit glial activation and reduce level cytokines (e.g., Il-6 blockers) and repair normal pain processing may be potential disease-modifying therapeutic options. Therapies that reduce neuroinflammation also could reduce reliance on polypharmacy, minimizing the burden of side effects from multiple medications. As research progresses, neuroinflammation targeting therapies could become a basis for personalized approaches in FM management.

### 2.4. Somatic and Psychological Aspects

Physical trauma, such as surgery, traffic accidents, certain infections, or experiences of war, can act as triggers for chronic pain and the onset of fibromyalgia [65,66,67]. A cohort study by Hunskar et al. revealed that individuals exposed to *Giardia lamblia* had a threefold higher prevalence of FM compared to those unexposed [68].

Psychiatric comorbidities also play a significant role in the development of FM. Multiple studies have reported that the prevalence of anxiety disorders and depression among FM patients in certain populations ranges from 30% to 60% [69]. Furthermore, patients with depressive symptoms tend to experience more intense and prolonged pain, as well as higher degrees of hyperalgesia and allodynia, compared to healthy controls. This suggests that depressive patterns may be associated with a worse prognosis in FM [70]. Genetic factors also appear to contribute to both FM and depression. For example, the serotonin transporter promoter region (5-HTTLPR) polymorphism, associated with anxiety-related personality traits, has been identified in individuals with FM [71].

Stress, much like depression, is both a predictive factor and a negative prognostic indicator for FM. Stress has been shown to influence pain sensitivity by inducing hyperalgesia or allodynia through mechanisms such as HPA axis dysregulation, glial activation, proinflammatory cytokine release, and the initiation of the pathological processes outlined above. Similar mechanisms are implicated in the pathogenesis of depression [72].

Patients with FM very often report sleep disturbances with a frequency of about 90% of patients [73]. It is interesting and very important to emphasize that sleep disorders do not necessarily have to be a symptom of this disease but could instead be causative factors. [73]. Studies indicate a bidirectional relationship between sleep disturbances and widespread musculoskeletal pain, with insomnia often preceding the onset of pain and serving as a predictor of its development and persistence [74]. The interruption of delta activity and slow-wave sleep by alpha activity reduces the pain-relieving effects of synaptic downscaling, thereby lowering the musculoskeletal pain threshold and contributing to fatigue and low energy levels [75,76,77].

Sleep disturbances are also associated with the exacerbation of various FM symptoms, including increased pain sensitivity at tender points, depression, and fatigue. Sleep plays a critical role in the pain pathway by activating the descending inhibitory pain mechanisms [76,77,78,79,80].

Studies involving healthy individuals have demonstrated that complete, partial, or specific types of sleep deprivation can lead to hyperalgesia, a higher incidence of spontaneous pain, and mood disturbances, particularly anxiety and depression [81,82]

**Figure 1:**
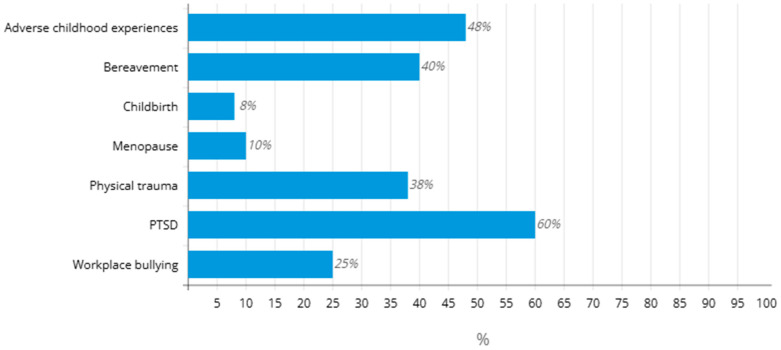
Patient respondent’s perception of causal factors responsible for Fibromyalgia [84].

The patient’s perspective is a critical factor in determining treatment adherence and outcomes, especially for chronic and complex conditions like FM. This perspective, or rather perception, of the causal factors is shown in the Figure 1. Understanding and integrating a patient’s beliefs, experience, and preferences into their care plan can significantly impact their willingness to follow therapy recommendations and the effectiveness of such treatment. Patients who understand FM as a manageable condition are more likely to actively participate in their care, unlike those who view FM as an untreatable disorder. Clear communication between patients and health providers about the realistic goals of treatment is essential. Unrealistic expectations can lead to frustration and the abandonment of the treatment program. Furthermore, a lack of understanding or support from family or friends may discourage adherence [83].

## 3. Diagnostics of Fibromyalgia and Classification Criteria

All previous research on fibromyalgia in more than 30 years has enabled the publication of at least five different classifications and sets of diagnostic criteria for FM. Based on the earliest set of criteria, fibromyalgia was described as a widespread pain condition with a variety of associated symptoms.

**Figure 2:**
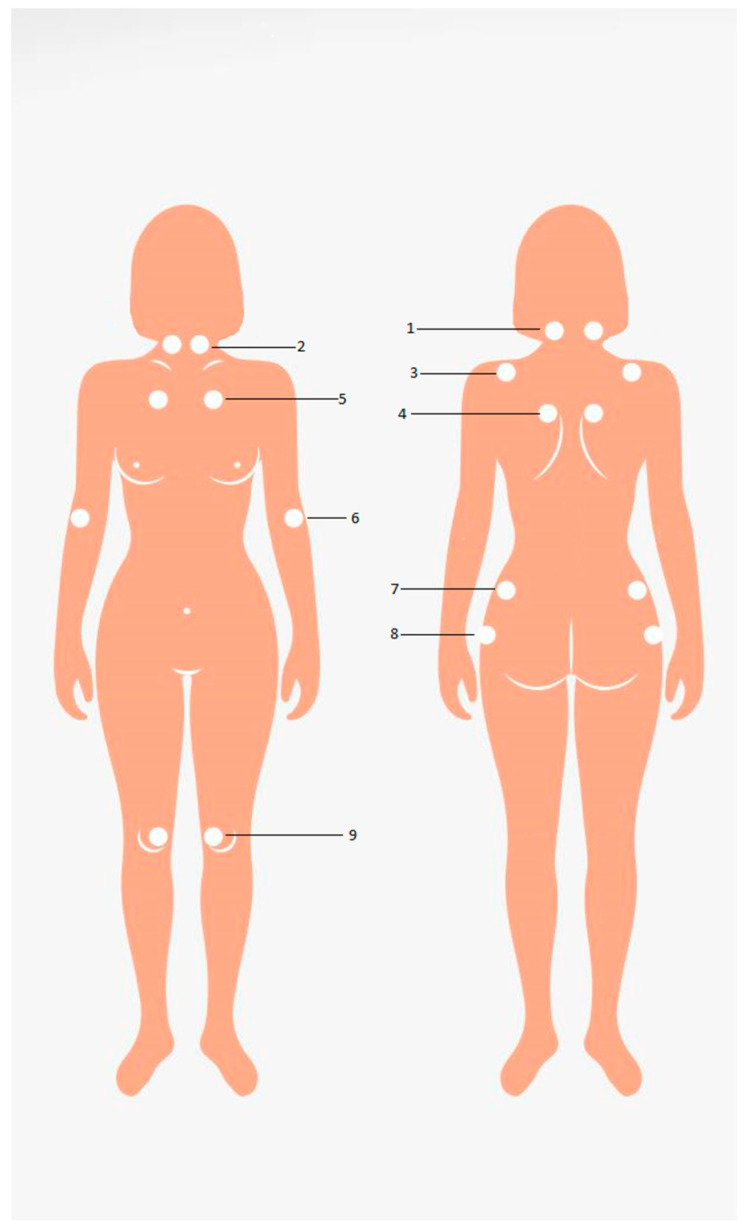
American College of Rheumatology (ACR) tender points for Fibromyalgia [85]. 1. Occipital: on both sides at the point of attachment of the suboccipital muscles. 2. Cervical: on both sides at the level of intertransferal spaces between C5 and C7 from the front. 3. Trapezius: bilaterally in the middle of its upper edge. 4. Supraspinatus: bilaterally above the scapula near the midline. 5. Second rib: bilaterally at the 2nd costochondral junction. 6. External epicondyle: bilaterally, 2 cm distally from the epicondyle. 7. Gluteal: bilaterally in the upper outer quadrant. 8. Greater trochanter: bilaterally from the back trochanter. 9. Knees: both sides on the inside, proximal to the joint line.

In 1990, the American Association of Rheumatologists (ACR) published classification criteria that gave the greatest importance to generalized pain. The same group of authors defined pain in FM as broad and widespread if it is present in the left and right half of the body, above and below the waist, with the presence of pain in the axial skeleton (cervical spine, front chest wall, thoracic and lumbar spine), for a continuous duration of at least 3 months. Clinical confirmation implies the presence of pain in 11 out of 18 sensitive, clearly defined points called tender points as seen in Figure 2.

A clinical diagnosis of fibromyalgia is made when both criteria are met: the presence of widespread pain that has been continuously present for at least 3 months, with pain on finger pressure in 11 of 18 tender points [85], and not ‘trigger points”’ as is sometimes commonly used in clinical, everyday practice. Difference between trigger and tender points are shown in Table 1. The presence of another disease does not exclude the diagnosis of FM.

In order to avoid the subjectivity of the doctor and the patient in making diagnoses, the diagnostic criteria have been revised. Revisions of the classification in 2010 and 2011 reduced the importance and palpability of tender points. In 2016, the American Association of Rheumatology introduced new diagnostic criteria with the aim of minimizing the possibility of misdiagnosis, but their reliability depends on several factors [88]. The criteria focus on the existence of generalized pain and the presence of comorbidities, especially fatigue and cognitive disorders. To establish a diagnosis of fibromyalgia, the following criteria must be met: WPI (Widespread Pain Index) ≥ 7 i SSS (Symptom Severity Score) ≥ 5 or WPI 4-6 i SSSS ≥ 9, the presence of generalized pain (defined as pain in at least four out of five regions left and right upper region, left and right lower region and axial region, while pain in the jaw, chest, and stomach does not belong to the definition of generalized pain), and that the symptoms have been present for at least 3 months. Moving away from tender point examinations, which were subjective and difficult to standardize, and focusing on the patient’s overall experience of pain and associated symptoms increases the criteria’s reliability. Additionally, using WPI and SSS scales reduces variability in diagnosis. This set of criteria can be used by nonspecialists and does not require the specialized physical examination of tender points, which improves their accessibility. On the other hand, there are some challenges and limitations. First, patients may overestimate, underestimate, or describe their symptoms differently, leading to potential misdiagnosis. Symptoms like fatigue and cognitive issues could lead to an overdiagnosis, especially in patients with depression or anxiety. Failure to rule out other conditions can lead to false positives.

The same criteria underlie the Analgesic, Anesthetic, and Addiction Clinical Trial Translations Innovations Opportunities and Networks—American Pain Society Pain Taxonomy (ACTTION) set of criteria from the year 2018, which singled out sleep disorders and fatigue, but also sensitivity to factors of the external environment: cold, light or noise, sensitivity to touch, in order for criteria to be more practical [89]. Evolution of diagnostic criteria of FM is presented in Table 2.

Fibromyalgia lacks specific biomarkers and laboratory tests, making it a diagnosis of exclusion. This can lead to variability between the clinicians. Some patients with FM may not meet strict diagnostic criteria, leading to underdiagnosis and vice versa. Overreliance on self-reported symptoms can lead to overdiagnosis. This is particularly found in cases where other conditions (e.g., rheumatoid arthritis, systemic lupus, psychiatric condition) are not correctly ruled out, even with a detailed evaluation of the medical history and the use of additional analyses, tests, and imaging.

Unlike other rheumatic diseases, FM does not manifest itself with visible clinical signs, so a clinical examination will reveal an increased sensitivity to pressure, which patients will describe as pain in certain points or regions of the body. The pain described by patients often resembles neuropathic pain, so 20 to 30% of patients report burning and tingling in the extremities, trunk, or hands. In addition to generalized pain, which is the cardinal symptom of FM, cognitive dysfunctions can also occur, especially ‘fibro fog’, i.e., memory defects, then depression, anxiety, and sleep problems. Patients with FM often complain of symptoms originating from any organ and organ system: headaches, more frequent migraines, dyspepsia, abdominal pain, constipation, diarrhea, urgency to urinate in the absence of a urinary infection, dysmenorrhea, stiffness, mostly morning stiffness lasting less than 60 min, and restless leg syndrome.

In identifying a patient who is at risk of developing FM, a number of screening tests have been developed that can be helpful to the physician in clinical work Fibromyalgia Rapid Screening Tool consisting of six questions, the FibroDetect test [90], as well as the assessment questionnaires of disease severity and effects of therapy: Fibromyalgia Impact Questionnaire (FIQ) and its modified version (FIQR) [91], the Fibromyalgia Survey Criteria (FSC), and the Fibromyalgia Assessment Status (FAS) [92,93].

## 4. Therapeutic Approach

Genetic predisposition, personal experiences, emotional-cognitive status, mind-body relationship, and tolerance to stress in their own unique way contribute to the development of fibromyalgia in each patient, which is why this disease is defined as psychosomatic.

The heterogeneity of FM significantly impacts both diagnosis and treatment, as it presents a wide spectrum of symptoms that vary in severity, combinations, and underlying contributing factors. This variability is a challenge for clinicians and requires a holistic, comprehensive, multidisciplinary, patient-centered approach. In summary, a more individualized approach to management. According to the EULAR recommendations [94], from 2016, treatment begins with patient education and involves the simultaneous application of pharmacological and nonpharmacological treatments. If there is insufficient effectiveness of one treatment modality, the therapeutic approach should be modified and adapted to the needs of the patient. Primarily, it is essential to emphasize that the heterogeneity of FM means that treatments effective for one patient may be ineffective or even counterproductive for another. Due to differences in pain threshold, psychological factors, and comorbidities, patients may respond differently to the same interventions.

### 4.1. Pharmacotherapy

**Figure 3:**
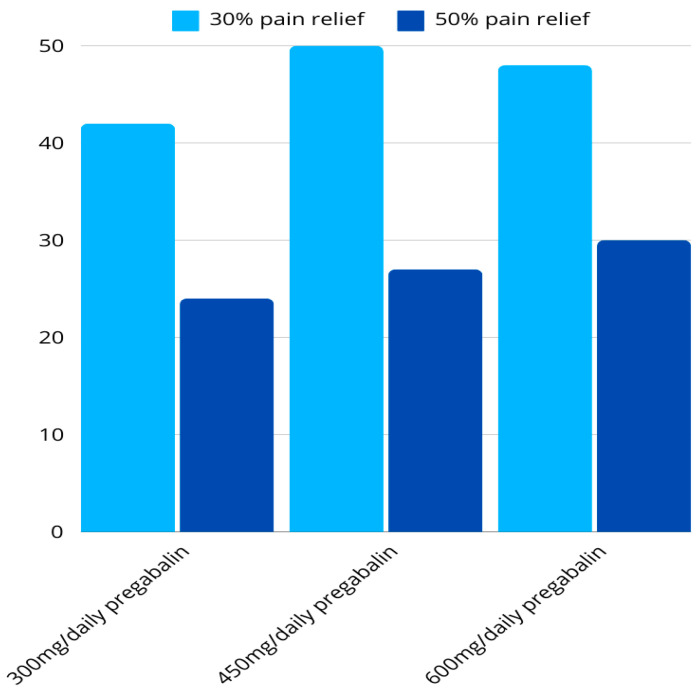
Efficiency of 300, 450, and 600 mg/daily of pregabalin in 30% and 50% pain relief in patients with Fibromyalgia [99].

Pharmacological treatment should be focused on the type of pain and the mechanism of its occurrence, which is why drugs that have a central action are those that show the highest degree of effectiveness. In addition to reducing pain, medications can alleviate other symptoms that affect the quality of life of patients with FM, such as sleep disorders, fatigue, anxiety, or depression. The most commonly prescribed drugs in the treatment of FM are antidepressants: tricyclic antidepressants (amitriptyline and nortriptyline), which can be useful in improving the quality of sleep (level of evidence Ia), selective serotonin reuptake inhibitors (SSRIs) such as duloxetine and sertraline(Ib)—especially if there are symptoms of depression or anxiety—as well as selective inhibitors uptake of serotonin and noradrenaline (SNRI)(Ia) [95]. Anticonvulsants (pregabalin and gabapentin) are the drugs whose effect in the treatment of FM has been more extensively investigated, and its efficiency is shown in the Figure 3. Pregabalin is currently the only anticonvulsant approved by the Food and drug administration (FDA) for the treatment of FM despite many side effects, such as dizziness (Ia) [95,96]. Certain therapy, such as opioids and non-steroidal anti-inflammatory drugs (NSAIDs), is not recommended in the treatment of FM due to the risk, lack of efficiency, or concerns about long-term consequences. The only opioid that could be somewhat effective is tramadol, with or without paracetamol [97,98].

### 4.2. Nonpharmacological Therapy

The nonpharmacological approach includes a wide spectrum of alternative and complementary modalities, of which cognitive-behavioral therapy stands out as the most common and most studied type of psychotherapy for patients with FM [100]. It aims to help patients recognize inadequate patterns of thought and behavior in order to develop effective strategies to deal with stress, pain, and negative emotions that have been shown to lead to the worsening of symptoms. Mindfulness therapy has also been shown to be effective in the treatment of FM and is based on accepting one’s own condition, thoughts, and suffering without judgment. The next important step in the treatment of patients with FM is educating the patient to understand that FM is a real condition, which is functionally disabling but not progressive, and that there is no peripheral tissue damage. Patients should be shown the role they play in their own treatment, familiarized with the benefits of psychotherapy and various relaxation techniques, and clearly pointed out that adaptation to stress and increased resilience are necessary for further treatment. The latest EULAR recommendations are shown in Table 3. favor exercise therapy because aerobic training, as in the case of other chronic pain conditions, is an important disease-modifying factor. The role of the physiatrist in the treatment of patients with fibromyalgia is significant and aims to include patients in an appropriate, individually dosed exercise program. Aerobic training is highly recommended in patients with FM in order to reduce pain and improve psychophysical condition [101,102]. Research suggests that acupuncture may help reduce pain and improve function in patients with fibromyalgia and is recommended (albeit weakly) by EULAR due to the moderate quality of evidence in the literature. Hypnosis therapy has become interesting for research in the last few years due to its effect on reducing pain and improving sleep. Tai Chi, Qigong, and Yoga, based on physical movement with adequate breathing and mental relaxation, are promising and safe alternatives to conventional exercise.

There are studies dealing with new approaches in the treatment of fibromyalgia, such as the application of noninvasive brain stimulation techniques, which have been proven to reduce pain in a significant number of fibromyalgia patients. Recent meta-analyses have investigated the efficiency of neuromodulation techniques, particularly of repetitive transcranial magnetic stimulation (rTMS) and transcranial direct current stimulation (tDCS) in the treatment of FM. The meta-analysis by Sun et al. [103] highlighted that high-frequency rTMS targeting the left primary motor cortex (M1) is particularly effective in reducing pain intensity and improving the quality of life. Their study emphasized the importance of stimulation parameters, including frequency and target area, in achieving optimal outcomes. A systematic review and meta-analysis by Moshfeghinia et al. [104] evaluated the impact of tDCS on pain intensity in fibromyalgia patients. The analysis, which included 20 studies, revealed that active tDCS significantly reduces pain intensity compared to sham stimulation. The left M1 area was the most common stimulation target, and a 2 mA intensity was frequently used. Findings from two more studies suggest that both rTMS and tDCS are promising noninvasive neuromodulation techniques for managing fibromyalgia symptoms, particularly in reducing pain and improving the quality of life. However, the effectiveness can vary based on stimulation parameters such as frequency, intensity, target area, and session duration. Further research is necessary to establish standardized protocols and to explore the long-term benefits and safety of these interventions [103,104,105,106].

The patient’s perspective is crucial in managing fibromyalgia, a condition deeply influenced by subjective experiences and multifaceted symptoms. Integrating their fears, beliefs, unique capabilities, and goals into the treatment process fosters trust, improves adherence, and leads to better overall outcomes. By adopting a patient-centered care approach, which requires strong communication and listening skills, empathy, and flexibility to adjust to each patient’s specific needs, healthcare providers can help FM patients achieve not only symptom relief but also a greater sense of control over their condition. Implementation of a multidisciplinary approach often poses some challenges, particularly in low-resource settings. Lack of funding to hire and sustain a multidisciplinary team, limited physical infrastructure, such as space for rehabilitation facilities, and a weak health system. Patients and communities may resist the concept of a team-based approach due to mistrust of the healthcare system or a preference for traditional healing methods. In low-resource settings, the focus is often on addressing acute, life-threatening conditions, leaving little attention to chronic conditions, rehabilitation, and mental health. Future research direction for FM addressing the complexities of this condition and improving diagnostic and treatment approaches. Identifying subtypes of FM could lead to a more personalized approach and improved outcomes, as well as developing specific biomarkers that reflect FM subtypes or undelaying mechanisms. Understanding how genetic variations affect drug metabolism and response could lead to personalized treatment, minimizing side effects. It is necessary to conduct long-term studies to better understand the natural course of FM.

## 5. Conclusions

Fibromyalgia is a complex chronic pain condition and, as such, requires a comprehensive approach to diagnosis and treatment. Patients suffering from fibromyalgia have a problem with the central processing of pain and struggle with neuropathic pain. Although the exact causes of the disease are unknown, it is believed to be due to a combination of genetic and environmental factors. Other potential causes of FM may be related to infection, physical trauma, emotional stress, endocrine disorders, as well as immune activation. While the pathogenesis of fibromyalgia is still the topic of researchers, there are no specific biomarkers or laboratory tests, and the diagnosis is clinical.

Newer research, such as certain polymorphisms and gene expression, may indicate a predisposition to fibromyalgia. Modern therapy for fibromyalgia requires multidisciplinary, patient-centered treatment and includes a combination of pharmacological and nonpharmacological options. Subjective experiences and the patient’s perspective are crucial in managing fibromyalgia. Their beliefs, fears, trust in treatment, and support from family members are critical factors in determining treatment adherence and the success of therapy. Further research, new knowledge about the mechanism of origin, the discovery of disease biomarkers or FM subtypes, and new treatment modalities are necessary in order to shift from symptom-based therapy to addressing the root causes of FM.

## Figures and Tables

**Table 1:** Similarities and differences of Tender points and Trigger points [86,87].

Table 1	Tender Points	Trigger Points
Definition	Pain-sensitive points in the muscles, the junction of muscles—tendons, bursae or fat pads	Areas of muscle that are painful to the touch and have stiff (short) muscle fibers that create a pattern of pain referrals
Distribution	Symmetric widespread	Focal/asymmetric, any muscle
Allodynia/Hyperalgesia	Present in tender points and in control sites throughout the body	Present in the trigger point only
Treatment	Lifestyle modification, exercise, stretching, cognitive behavioral therapy, acupuncture, acupressure, massage, yoga pharmacotherapy for Fibromyalgia	Transcutaneous Electrical Nerve Stimulation (TENS), dry needling, high-intensity focused ultrasound, pressure release, ice pack/warm pad, injection to the trigger point

**Table 2:** Evolution of diagnostic criteria of Fibromyalgia.

Authority/Organization	Diagnostic Criteria	Key Updates	Associated Symptoms
American College of Rheumatology (ACR)	ACR 1990 Criteria	First standardized diagnostic criteria. Focused on tender points and widespread pain.	Chronic widespread pain, localized tenderness, morning stiffness, fatigue.
	- Widespread pain for at least 3 months.		
	- Pain on palpation of 11 out of 18 specific tender points.		
ACR	ACR 2010 Preliminary Criteria	Emphasis on a broader range of symptoms (e.g., fatigue, cognitive issues).	Chronic pain, fatigue, sleep disturbances, cognitive dysfunction (“fibro fog”), and headaches.
	- Removed tender point exam.		
	- Introduced Widespread Pain Index (WPI) and Symptom Severity Scale (SSS).		
ACR (Modified)	2011 Modified ACR Criteria	Allowed diagnosis in clinical practice without tender point examination.	Chronic pain, stiffness, fatigue, memory issues, depression, anxiety, and irritable bowel symptoms.
	- Simplified the 2010 criteria.		
	- Introduced self-report surveys for WPI and SSS.		
ACR	Revised ACR 2016 Criteria	Improved specificity and reduced overdiagnosis.	Widespread pain, unrefreshing sleep, fatigue, sensitivity to external stimuli (light, noise), depression.
	- Added a generalized pain criterion (at least 4 of 5 regions).		
	- Retained WPI and SSS but refined scoring rules.		
European League Against Rheumatism (EULAR)	EULAR 2019 Guidelines	Provided a comprehensive approach for diagnosis and treatment, not solely criteria-based.	Pain amplification, severe fatigue, sleep disorders, mood disorders, cognitive impairments, and IBS.
	- Emphasized multidisciplinary management of FM.		
	- Highlighted the importance of patient-reported outcomes and functional impact.		
Research and Clinical Practice	Evolving understanding of FM as a centralized pain condition with possible genetic, psychological, and environmental influences.	New biomarkers and imaging technologies are under investigation to enhance diagnosis.	Neuroinflammation, altered pain pathways, chronic fatigue, anxiety, depression, and autonomic issues.

ACR—American College of Rheumatology; FM—Fibromyalgia.

**Table 3:** European League Against Rheumatism (EULAR) Fibromyalgia (FM) therapy recommendations [94].

Treatment	Mechanism of Action	Evidence Level (EULAR)	Potential Side Effects
Amitriptyline	Increases serotonin and norepinephrine levels to modulate pain pathways	IA (low-dose only)	Sedation, dry mouth, weight gain, dizziness
Pregabalin	Reduces nerve signaling related to pain via calcium channel modulation	IA	Dizziness, somnolence, weight gain, peripheral edema
Duloxetine	Inhibits serotonin and norepinephrine reuptake to enhance pain modulation	IA	Nausea, headache, dry mouth, fatigue
Cognitive Behavioral Therapy (CBT)	Addresses negative thought patterns and coping mechanisms for pain management	IA	None specific; potential emotional discomfort during therapy
Exercise (Aerobic/Strength Training)	Enhances endorphin release and improves overall physical functioning	IA	Muscle soreness, risk of overexertion
Mindfulness-based Stress Reduction (MBSR)	Reduces stress and enhances self-regulation of pain	IA	None specific; initial frustration or difficulty adhering to practice
Acupuncture	Stimulates nerve pathways and alters pain perception	IA	Mild bruising, temporary pain at needle sites

CBT—Cognitive Behavioral Therapy; MBSR—Mindfulness-based Stress Reduction; EULAR—European League Against Rheumatism.
